# Supplementary material for: Magnetic field controlled charge density wave coupling in underdoped YBa2Cu3O6+x
Source: Nat Commun. 2016 May 5;7:11494. doi: 10.1038/ncomms11494 (PMC4858734; doi:10.1038/ncomms11494)
Supplement: Supplementary Information — Supplementary Figures 1-5, Supplementary Tables 1-2, Supplementary Notes 1-4 and Supplementary References [file ncomms11494-s1.pdf]

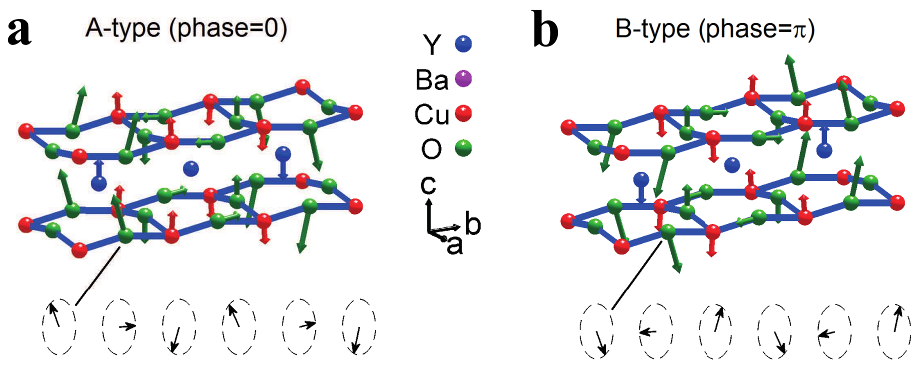

Supplementary Figure 1: CDW structure within single unit cells (bilayers) of type-A ($\phi_c = 0$) and type-B ($\phi_c = \pi$). Only atoms in and between the CuO_2 planes are shown for clarity; these have the largest displacements. The arrows represent the exaggerated displacements of the atoms. The ellipses with arrows represent the displacements of the planar oxygen atoms as a function of position along the b-axis.

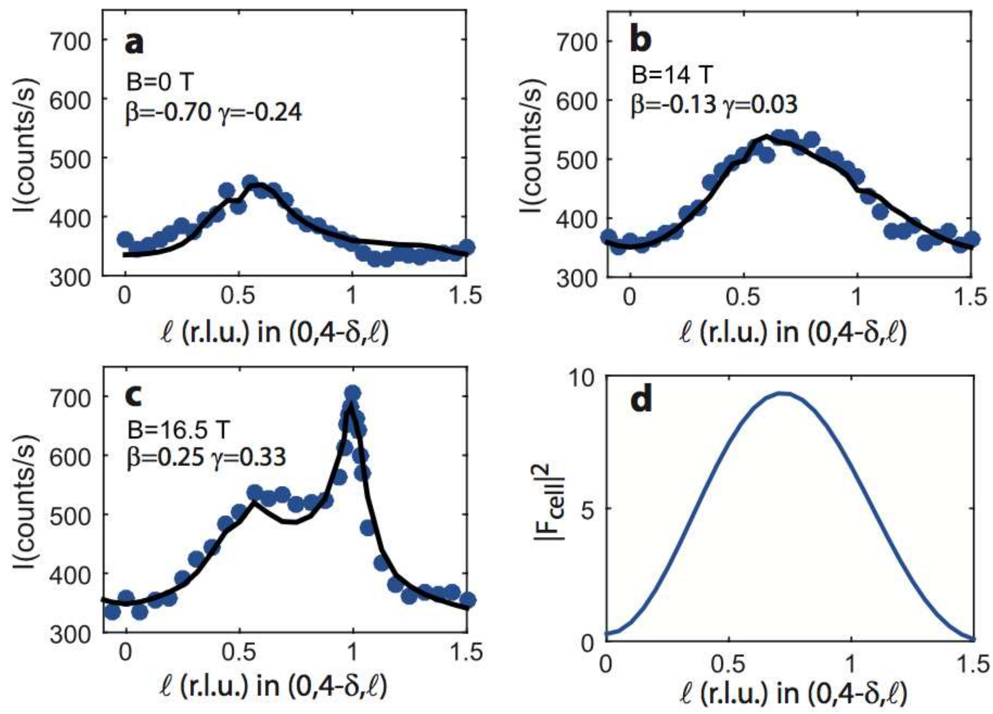

Supplementary Figure 2: (a)-(c) Markov-generated profiles fitted, with a constant background added, to data such as that in Fig. 1 of the main text. Data collected at $T \approx 8$ K on $YBCO_{6.67}$. (d) CDW structure factor $|F_{\rm cell}|^2$ for a single unit cell (F in arbitrary units).

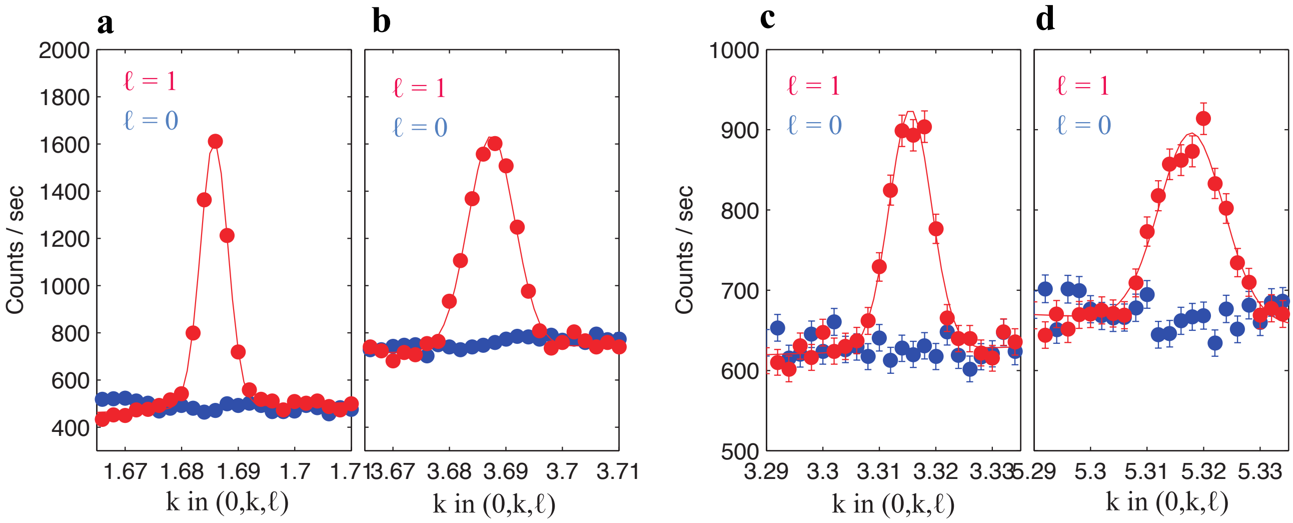

Supplementary Figure 3: Compilation of k-scans, recorded on $YBCO_{6.67}$ at 16.5 T and 8 K, through selected $(0, n \pm \delta_b, \ell)$ positions with $\ell = 0$ (blue) or 1 (red). No background subtraction has been made. The instrumental resolution gradually increases with $|Q| = (h^2 + k^2 + l^2)^{0.5}$ leading to broader CDW peaks. A compilation of integrated Bragg peak intensities is given in Supplementary Table 1. Error bars are given by counting statistics.

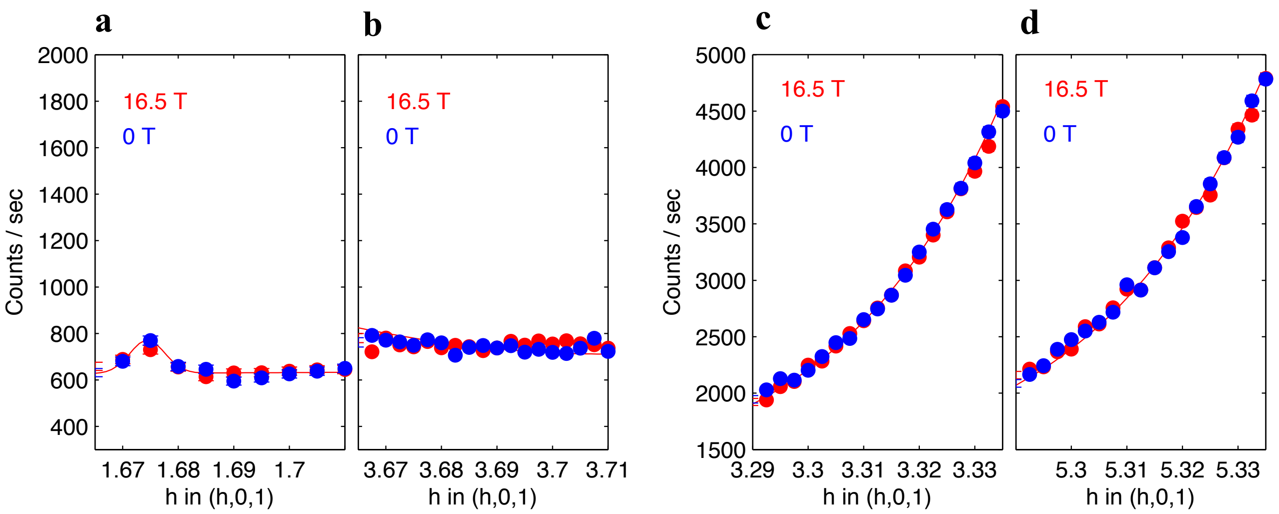

Supplementary Figure 4: h-scans through selected $(n \pm \delta_a, 0, 1)$ positions in $YBCO_{6.67}$ at 16.5 T (red) and zero-field (blue) for $T = 8$ K. No background subtraction has been made. Within statistical errors, no field-induced signal is observed. All scanned positions with $\ell = 0, 1$ and 2 are indicated in Supplementary Table 1. Error bars are determined by counting statistics.

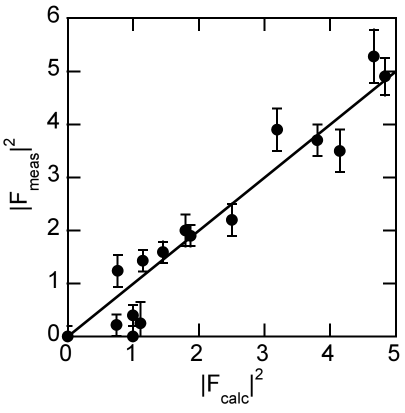

Supplementary Figure 5: A comparison of the measured CDW structure factors of the 3D CDW peak observed at high fields with those calculated from the Δ_3 structure described in Sec. IV. In this structure, the values of the atomic displacements within the unit cell have been taken to be the same as the average zero-field structure - except for those in the CuO chains, which have been set to zero, since their b-axis displacements are zero by symmetry and any c-axis displacements have a very small effect at low ℓ. The theoretical F values are in arbitrary units, and the experimental values are derived directly from measured intensities. A single multiplying factor has been used to fit the experimental data to the theoretical values.

$(0,k,\ell)$	$\ell=0$	$\ell=0.5$	$\ell=1$	$\ell=2$
$k=2-\delta_b$	0 ± 0.02	1.95 ± 0.34	3.9 ± 0.2	-
$k=2+\delta_b$	0 ± 0.02	0.63 ± 0.23	2.2 ± 0.1	-
$k=3-\delta_b$	0 ± 0.03	1.75 ± 0.5	1.43 ± 0.15	-
$k=3+\delta_b$	0 ± 0.03	spurions	1.59 ± 0.14	-
$k=4-\delta_b$	0 ± 0.03	2.6 ± 0.4	5.28 ± 0.25	0 ± 0.1
$k=4+\delta_b$	0 ± 0.06	1.3 ± 0.2	3.7 ± 0.3	0.4 ± 0.2
$k=5-\delta_b$	0 ± 0.07	0.88 ± 0.4	2.0 ± 0.3	0.22 ± 0.1
$k=5+\delta_b$	0 ± 0.03	0.29 ± 0.28	1.9 ± 0.2	1.24 ± 0.2
$k=6-\delta_b$	0 ± 0.09	2.2 ± 0.03	4.9 ± 0.35	0.25 ± 0.4
$k=6+\delta_b$	0 ± 0.1	Not measured	3.5 ± 0.4	Not measured
$(h,0,\ell)$	$\ell=0$	$\ell=0.5$	$\ell=1$	$\ell=2$
$h=2-\delta_a$	spurions	5.8 ± 0.6	0 ± 0.2	-
$h=2+\delta_a$	0 ± 1.5	Not measured	0 ± 0.25	-
$h=3-\delta_a$	0 ± 0.5	Not measured	0 ± 0.25	-
$h=3+\delta_a$	0 ± 1.2	Not measured	0 ± 0.4	-
$h=4-\delta_a$	0 ± 0.5	15 ± 3	0 ± 0.2	0 ± 1.5
$h=4+\delta_a$	0 ± 1.0	Not measured	0 ± 0.3	0 ± 0.5
$h=5-\delta_a$	0 ± 1.0	Not measured	0 ± 0.4	0 ± 1
$h=5+\delta_a$	0 ± 1.5	Not measured	0 ± 0.3	0 ± 0.5
$h=6-\delta_a$	0 ± 1.5	Not measured	0 ± 0.3	0 ± 0.5
$h=6+\delta_a$	0 ± 0.8	Not measured	0 ± 0.3	Not measured

Supplementary Table 1: Integrated intensity (arbitrary units) obtained, at 16.5 T, by fitting k- and h-scans on $YBCO_{6.67}$ to a single Gaussian function as exemplified in Supplementary Figure 3 for $\ell=0$ and 1. "0" indicates no visible peak and no field induced signal. The term "spurions" is used for peaks in the scan range that were found to have no temperature dependence.

$(h,0,\ell)$	$\ell=0$	$\ell=0.5$	$\ell=1$	$\ell=2$
$h=2-\delta_a$	spurions	0.28 ± 0.14	Not measured	Not measured
$h=2+\delta_a$	spurions	0.33 ± 0.08	0 ± 0.06	Not measured
$h=4-\delta_a$	0 ± 0.07	0.32 ± 0.1	0 ± 0.06	Not measured
$h=4+\delta_a$	spurions	0.3 ± 0.08	0 ± 0.05	Not measured
(h,k,ℓ)	$\ell=0$	$\ell=0.5$	$\ell=1$	$\ell=2$
$h=2,k=2-\delta_b$	Not measured	0.13 ± 0.06	0.45 ± 0.04	Not measured
$h=2+\delta_a,k=2$	Not measured	0.33 ± 0.1	spurions	Not measured

Supplementary Table 2: Integrated intensity (arbitrary units) obtain by fitting h-scans recorded on $YBCO_{6.60}$ ortho-II at 16.9 T.

I. SUPPLEMENTARY NOTE 1: CDW STRUCTURE OF A SINGLE BILAYER

A CDW implies a periodic modulation of the atomic position of each atom j in the unit cell. It has a characteristic wavevector $\mathbf{q}$. We may write the positions $\mathbf{r}_j$ of the atoms in the modulated structure in terms of their regular positions $\mathbf{r}_\mathrm{j}^0$ and the displacement parameters $(\mathbf{u}_\mathrm{j}', \mathbf{u}_j'')$ of the CDW as:

$$\mathbf{r}_\mathrm{j} = \mathbf{r}_\mathrm{j}^0 + \mathbf{u}_\mathrm{j}' \cos\left(\mathbf{q}\cdot\mathbf{r}_\mathrm{j}^0 + \phi_\mathrm{c}\right) + \mathbf{u}_\mathrm{j}'' \sin\left(\mathbf{q}\cdot\mathbf{r}_\mathrm{j}^0 + \phi_\mathrm{c}\right), \tag{1}$$

where we have included an arbitrary phase ϕ_c, which would be a constant throughout the crystal for long-range order. As discussed in the main text, the CDW in $YBa_2Cu_3O_{6+x}$ is somewhat disordered, with a short coherence length in the c-direction, leading to CDW satellite peaks with a large width in ℓ and centred approximately at positions $\ell = n + \frac{1}{2}$ with integer n. By assuming that CDW has a periodicity of $2c$ along the c-axis, the average structure in zero field and at T_c can be solved [1] and the displacement parameters $\mathbf{u}_\mathrm{j}'$ and $\mathbf{u}_\mathrm{j}''$ determined. The atoms near the CuO_2 bilayers have the largest amplitude displacements. This approach [1] avoids an explicit description of the c-axis disorder, and the CDW structures have wavevectors $\mathbf{q}_\mathrm{a}$=(δ_a,0,1/2) and $\mathbf{q}_\mathrm{b}$=(0,δ_b,1/2). The half-integral ℓ in these wavevectors corresponds to a phase difference of π between the arguments $(\mathbf{q}\cdot\mathbf{r}_\mathrm{j}^0 + \phi_\mathrm{c})$ of the trigonometric functions in Supplementary Equation 1 for equivalent atoms in neighbouring unit cells (bilayers) along the c-axis. An alternative approach is to remove the $\ell = 1/2$ components from $\mathbf{q}$ so that $\mathbf{q}_\mathrm{a}$ and $\mathbf{q}_\mathrm{b}$ have basal plane components only, and the variation along the c-direction is represented by position-dependent values of ϕ_c. In this case, we can describe the CDW structure in terms of a stacking sequence of two types of bilayer: A-type with ϕ_c= 0 and B-type with ϕ_c= π (see Supplementary Figure 1). The average low-field structure would then have the stacking sequence along the c-axis of ... ABABABA ... and we represent the disorder by imperfect stacking sequences.

II. SUPPLEMENTARY NOTE 2: RESULTS OF MARKOV MODELING

The 3D structure of the CDW in $YBa_2Cu_3O_{6+x}$, due to a modulation component $\mathbf{q}_\mathrm{a}$or $\mathbf{q}_\mathrm{b}$, can be visualised as a stacking sequence (e.g. ABBAABA) of unit cells (bilayers) of type-A or type-B described above and shown in Supplementary Figure 1. Stochastic sequences of bilayer type (A/B) are generated using a memory $m = 2$ Markov chain with nearest-neighbour and next-nearest-neighbour parameters β and γ. The resulting scattering profiles are computed, averaged and fitted to the data. The profiles are corrected for the CDW structure factor $|F_\mathrm{cell}|^2$ (see Supplementary Figure 2(d)) calculated by summing over the displacements of atoms in the original unit cell ($a \times b \times c$) of the YBCO structure. The displacements used are determined in Ref. [1], including those along the a- or b-axes of the Cu and O atoms in the chain layers. This model gives low intensity near $\ell \approx 0$ and $\ell \approx 1.5$. It does not allow for changes in the chain layer modulation which will undoubtedly take place as the stacking sequence along c changes. Nevertheless, it provides a good description of the data with the results of the fitting shown in Fig. 2, and we have confirmed that the derived coupling parameters β and γ are highly insensitive to changes in the $|F_\mathrm{cell}|^2$ that would result from reasonable modifications of the chain layer displacements (e.g. setting them to zero).

III. SUPPLEMENTARY NOTE 3: CDW BRAGG PEAK INTENSITIES:

Our experimental setup at the P07 beamline gives access to the scattering planes $(h, 0, \ell)$, $(0, k, \ell)$ and (k, k, ℓ). The magnet windows (beam-in and beam-out) allow scattering angles up to $\sim$ 20 degrees, but for experiments with the applied field approximately parallel to the c-axis, ℓ is constrained to small values < 3.

The $\mathbf{q}_\mathrm{b}$= (0, δ_b, 0) CDW modulation was measured in many different Brillouin zones for $YBCO_{6.67}$. The results are listed in SupplementaryTable 1. Fig. 3 shows how $\mathbf{q}_\mathrm{b}$-CDW reflections are found at $\ell = 1$, but not at the equivalent $\ell = 0$ positions. We also investigated the a-axis direction where no CDW order was found at $\ell = 0$, 1, or 2, see Supplementary Figure 4 and Supplementary Table 1. However, there was a half-integral ℓ signal along the a-axis, which became enhanced with increasing field, but showed no sign of becoming fully 3-dimensional. We also found no evidence of CDW order with $\ell = 0$ or 1 along the a-axis direction in $YBCO_{6.60}$ (Supplementary Table 2).

IV. SUPPLEMENTARY NOTE 4: STRUCTURE OF THE 3D ORDER OBSERVED AT HIGH FIELD

Group theory [2] indicates that there are four possible symmetries $(\Delta_1, \Delta_2, \Delta_3, \Delta_4)$ for a CDW with incommensurate modulation of the form $\mathbf{q} = (0, \delta, 0)$. Δ_2 and Δ_4 are polarised along $\mathbf{a}$ and are not observed here. The Δ_3 mode has c-axis displacements that are equal and in the same direction in the two halves of a bilayer together with b-axis

displacements that are equal and opposite (See Supplementary Figure 1). The Δ_1 mode has the b- and c-axis bilayer symmetries interchanged. The bilayer symmetry corresponding to Δ_3 is that established in zero field [1]. We expect that the intra-bilayer interaction will maintain the phase relationship between the two halves of a bilayer to high field, and that the sole change will be in the c-axis stacking, controlled by the weaker inter-bilayer interaction. Thus the Δ_3 mode corresponds to an ... AAAAA ... stacking of the bilayer structure shown in Supplementary Figure 1. The contribution of an ionic displacement $\mathbf{u}$ to the CDW scattering amplitude for a satellite at wavevector $\mathbf{Q}$ is $\propto \mathbf{Q} \cdot \mathbf{u}$ (Ref. [1]). For $\ell = 0$, $\mathbf{Q}$ is perpendicular to the c-axis, so the c-axis displacements make zero contribution to the CDW satellite intensity. For the Δ_3 mode there are equal and opposite b-axis displacements at equivalent positions in the unit cell, so their contribution at all $\mathbf{Q}$s having $\ell = 0$ is also zero. For the Δ_1 CDW pattern allowed by group theory, this is not the case. Our observation of zero CDW intensity at $\ell = 0$ is therefore strong supporting evidence for the Δ_3 structure. We note that at nonzero ℓ, both types of displacement in this structure give nonzero contributions to the CDW scattering amplitude. We have modelled our set of integer-ℓ intensity measurements with atomic displacements for a Δ_3 mode closely related to the zero-field structure. Supplementary Figure 5 shows that this gives a good account of our observations, confirming the high-field structure. It should be noted that the 3D order whose structure is described above co-exists with other CDW correlations. In particular, the $\mathbf{q}_\mathrm{a}$ modulation shows weak anti-phase correlations between neighbouring bilayers at the highest fields $B = 16.9$ T investigated here.

V. REFERENCES

[1] Forgan, E. M. *et al.* The nature of the charge density waves in under-doped $YBa_2Cu_3O_{6.54}$ revealed by x-ray measurements of the ionic displacements. *Nat. Commun.* **6**, 10064 (2015).

[2] Campbell, B. J. *et al.* *ISODISPLACE*: A web-based tool for exploring structural distortions. *J. App. Crystal.* **39**, 607–614 (2006).
